# Surgical leadership: the British concept

**DOI:** 10.1515/iss-2019-0006

**Published:** 2019-05-10

**Authors:** John N. Primrose

**Affiliations:** FMedSci, Professor of Surgery, University of Southampton, Past President, Association of Surgeons of Great Britain and Ireland, University Surgery, Mailpoint 816, C Level South Academic Block, Southampton General Hospital, Southampton, UK, Phone: +44 (0) 23 8120 6144

**Keywords:** clinical service, college, leadership, management, specialty association

## Abstract

In the UK all surgeons need to have leadership skills as, despite the increasing importance of team management, the consultant surgeon still has overall responsibility for the patient under their care. Poor care is evident when clinical leadership fails. The UK hospital system is non-hierarchical within the consultant body. The clinical surgical manager in a hospital may not be the most senior clinician and the role will often rotate. The manager may or may not have the characteristics of a leader, and very often surgical leaders in a hospital may have no formal role. They are, however, essential to the functioning of the service. Nationally the roles in which professional leadership may reside are numerous. The country has multiple Surgical Royal Colleges and innumerable specialty associations and sub specialty associations all of which have councils and presidents and the multiple specialty and sub-specialty associations normally will have an annual meeting. In the long term this is probably unsustainable and although consolidation is desirable it is hard to achieve. In summary, good surgical leadership is found in many settings in the UK, some in formal roles within hospitals, some in the colleges and specialty associations and sometimes in individuals with no formal role but the capacity to make things happen.

*“Leadership is the art of getting someone else to do something you want done because he wants to do it”.* Dwight D. Eisenhower

In the UK all surgeons, whether or not they have a formal leadership role, are expected to demonstrate leadership skills, whether in the operating room, the hospital ward or being a leader in a clinical or academic service [1]. The model for surgical care has certainly changed over recent decades, from the autonomous consultant with subservient staff to a much more team-based approach. But one thing that has not changed is that patients have a named individual consultant surgeon whose name is “over the bed” and hence it is the surgeon who is ultimately responsible with the institution for the patient’s care.

It is clear to see the consequences when medical leadership as a whole breaks down. The biggest disaster in recent years in the UK National Health Service (NHS) concerned the poor quality of care at Mid Staffs Hospital [2] resulting in significant excess mortality (perhaps up to 1200 deaths) and the appalling treatment of patients, many elderly. Whilst the causes were complex, and beyond the scope of this article to describe, one of the major failures was the absence of any effective medical or surgical leadership. Leadership in clinical services is not optional if quality is to be maintained.

Having established a simple principle that leadership matters, a description of surgical leadership in the UK becomes, to say the least, complex. Indeed, if one were constructing a system within surgery now one would not arrive at the present system. But history is history. In this paper, I will attempt to describe the structures which exist in the clinical service and the postgraduate organisations and try and determine where surgical leadership does or does not exist. And if surgery in the UK has a voice, where does it reside.

It is appropriate to start with the clinical service. At a senior level the medical structure in the UK is flat and non-hierarchical and everyone at consultant level is essentially on the same grade. This differs from many of the systems in continental Europe. In the past, this represented autonomy in terms of patient care. This has radically changed and it is now normal for clinical support and mentorship to be provided for newly appointed consultants. It is, however, not mandated by the service and we still see disasters where either surgeons do not accent support or if an unsupportive environment does not provide it.

Medical management has been incorporated into the hospital system since the reforms in the NHS several decades ago. The Clinical Service Director will commonly be drawn from the consultant body. Seniority is not a prerequisite to hold a management role, nor indeed is the ability to do the job. Substantial training is not commonly provided and the role is not especially attractive as it does not normally come with a lot of power (in hospitals power lies at a higher level) but is afflicted with a lot of tedious administration. The advantage is that there is not really a possibility that a service can be stuck with a powerful director who may in theory exhibit varying degrees of malevolence and block change. And clearly there is a difference between “management” and “leadership”, the former being generally about control, the later more about inspiration, motivation, and common goals. Clinical services in the UK often have obvious leaders, but commonly without them being in any formal role.

Universities and hospitals are separate entities in the UK and postgraduate medical education, including surgical training, is the responsibility of a national body (Health Education England, with other bodies for the Devolved Administrations) and not the universities. This is probably regrettable as education is best delivered by an organisation whose function and expertise that is. In university hospitals, there is a relationship between the relevant university and the hospital with varying degrees of harmony (or not). Surgical academics may be wholly university employed, part university employed or in many cases hospital employed but with an honorary university position. Leadership in the university sector is easier to define and the assessment criteria clear, at last in respect of seniority. Progress in the university requires high quality research outputs (few if any surgical journal publications would be included) and substantial grant income. This is not especially easy for the practicing surgical academic but academic surgical leaders do achieve this. That being said, of the 1200 members of the UK Academy of Medical Sciences there are only 20 surgeons.

Moving to the national environment the situation becomes more eclectic and complex. We have a system of colleges, focussing on postgraduate education and assessment (amongst a multiplicity of other things) and the specialist associations which are membership organisations for the relevant surgical specialty, commonly organising post graduate events and conferences.

There is one Surgical Royal College in England (population 55 million), two in Scotland (population 5 million) and also one in the Republic of Ireland (population 5 million). It is perhaps odd to have a “Royal” College in a republic but there has never been appetite for change, quite the reverse. Whilst one may say why not have just one College (at least in the UK) it would be no exaggeration to say that Hell would freeze first. The Colleges are very ancient organisations. The Edinburgh College was founded in 1505. Each College has an elected Council and President, all previously set their own exams (and still do overseas) although gladly in the UK the surgical examinations are now all intercollegiate. In other ways, however, attempts to unite College functions have failed. Even though an external review was carried out suggesting that an executive intercollegiate body be formed this was never implemented.

Specialist associations are a more recent innovation. The oldest, the Association of Surgeons of Great Britain and Ireland (ASGBI), founded by Lord Moynihan is just under 100 years old. There are a total of nine specialist associations representing the specialties within UK surgery (the last specialty being vascular surgery that came out of general surgery). Many are relatively small. Indeed, general surgery and orthopaedics together amount to two thirds of all the surgical consultants in the UK. They all have a Council and a President and all run an annual meeting. Unlike the Colleges they do have a federated council meeting (the Federation of Surgical Speciality Associations) and yes, it also has a President.

So we now have 10 major nationally significant bodies all of which have Presidents/Leaders. However, it is not just that simple. Many of the defined surgical specialties have within them multiple other organisations. Within “General Surgery” (in the UK this is visceral surgery, breast and endocrine, transplantation, trauma, hernia, etc) there are a multiplicity of societies covering every specialist area imaginable and also some technique-based societies. And yes, most of these societies also have a Council and President. Its similar in orthopaedics where most joints have a society. From my own perspective, from the biased position of a Past President of the ASGBI, a degree of radical consolidation would be welcome.

So coming back to the subject of this paper, leadership as opposed to structures, there are a lot of potential positions in which surgeons may lead in their specialty although whether this always amounts true professional leadership is less clear. It may be that the multiplicity of voices, rather than a single voice for surgery is a disadvantage. It is the case that Government, at least in England, (the Devolved Administrations having control of healthcare) will usually go to the Royal College of Surgeons of England if advice is sought on surgical matters, which it not always is. On the other hand, Specialty Associations have very much driven the specialty agenda, to the extent that complex major surgery is centralised in large centres with substantial case volumes. For instance, centres doing hepato-pancreato-biliary (HPB) surgery, like Southampton, will typically undertake many hundreds of cancer resections a year. The outcomes in terms of peri-operative morbidity and mortality are extremely good in the UK. Such rationalisation was the result of true leadership, as centralisation is not easy to achieve and not popular with those who may lose a service.

In summary there is no one model for surgical leadership in the UK nor any one setting in which it resides. Good surgical leadership is found in many NHS hospitals, frequently in university hospitals and more often than not in the associated university itself. However, surgeons in some smaller hospitals have achieved global prominence through their leadership in the profession and through the development of innovative practice and outstanding clinical services. What is obvious however, is that within organisations leaders may actually have no formal role. They are the surgeons without whom things do not happen.

## Supplementary Material

The article (iss-2019-0006) offers reviewer assessments as supplementary material.

## References

[1] Surgical Leadership: A Guide to Best Practice. Royal College of Surgeons of England, 2014. https://www.rcseng.ac.uk/-/media/files/rcs/standards-and-research/gsp/surgical-leadership--a-guide-to-best-practice.pdf. Accessed 01.04.2019.

[2] The Mid Staffordshire NHS Foundation Trust Public Inquiry, Chaired by Robert Francis QC HM Stationary Office, 2013. https://webarchive.nationalarchives.gov.uk/20150407084231/http://www.midstaffspublicinquiry.com/report. Accessed 01.04.2019.

