# Supplementary material for: Surgical leadership: the British concept
Source: Innov Surg Sci. 2019 May 10;4(2):65–7. doi: 10.1515/iss-2019-0006 (PMC6754055; doi:10.1515/iss-2019-0006)
Supplement: Supplementary file 1 [file iss-4-20190006-s001.pdf]

Reviewer Assessment

John N. Primrose*

Surgical leadership: the British concept

https://doi.org/10.1515/iss-2019-0006
Received April 15, 2019; accepted April 24, 2019

***Corresponding author: John N. Primrose,** FMedSci, Professor of Surgery, University of Southampton, Past President, Association of Surgeons of Great Britain and Ireland, University Surgery, Mailpoint 816, C Level South Academic Block, Southampton General Hospital, Southampton, UK, Phone: +44 (0) 23 8120 6144, E-mail: j.n.primrose@soton.ac.uk

Reviewers' Comments to Original Submission

Reviewer 1: anonymous

Apr 23, 2019

**Reviewer Recommendation Term:**	Accept
**Overall Reviewer Manuscript Rating:**	70
Is the subject area appropriate for you?	5 - High/Yes
Does the title clearly reflect the paper's content?	5 - High/Yes
Does the abstract clearly reflect the paper's content?	5 - High/Yes
Do the keywords clearly reflect the paper's content?	5 - High/Yes
Does the introduction present the problem clearly?	5 - High/Yes
Are the results/conclusions justified?	5 - High/Yes
How comprehensive and up-to-date is the subject matter presented?	5 - High/Yes
How adequate is the data presentation?	N/A
Are units and terminology used correctly?	5 - High/Yes
Is the number of cases adequate?	N/A
Are the experimental methods/clinical studies adequate?	N/A
Is the length appropriate in relation to the content?	5 - High/Yes
Does the reader get new insights from the article?	5 - High/Yes
Please rate the practical significance.	5 - High/Yes
Please rate the accuracy of methods.	N/A
Please rate the statistical evaluation and quality control.	N/A
Please rate the appropriateness of the figures and tables.	N/A
Please rate the appropriateness of the references.	5 - High/Yes
Please evaluate the writing style and use of language.	5 - High/Yes
Please judge the overall scientific quality of the manuscript.	5 - High/Yes
Are you willing to review the revision of this manuscript?	No: Not necessary

Comments to Author:

Thanks for sharing your experience with delivering the basics of surgical leadership in the UK. Due to the increasing challenges through innovative and more and more complex surgical care, team management within interdisciplinary concepts, economic constraints, and shortage of surgical trainees and staff surgical leadership becomes a very important competence of surgeons in the wide range of their profession. We can learn a lot from the settings in your country, based on activities of the Colleges and special societies. So the paper fits very well to Innovative Surgical Sciences and should be accepted without any revisions.

Open Access. © 2019 Primrose J.N., published by De Gruyter. This work is licensed under the Creative Commons Attribution 4.0 Public License.

Reviewer 2: anonymous

Apr 15, 2019

**Reviewer Recommendation Term:**	Accept
**Overall Reviewer Manuscript Rating:**	80
Is the subject area appropriate for you?	5 - High/Yes
Does the title clearly reflect the paper's content?	5 - High/Yes
Does the abstract clearly reflect the paper's content?	4
Do the keywords clearly reflect the paper's content?	5 - High/Yes
Does the introduction present the problem clearly?	N/A
Are the results/conclusions justified?	4
How comprehensive and up-to-date is the subject matter presented?	5 - High/Yes
How adequate is the data presentation?	N/A
Are units and terminology used correctly?	N/A
Is the number of cases adequate?	N/A
Are the experimental methods/clinical studies adequate?	N/A
Is the length appropriate in relation to the content?	4
Does the reader get new insights from the article?	4
Please rate the practical significance.	4
Please rate the accuracy of methods.	N/A
Please rate the statistical evaluation and quality control.	N/A
Please rate the appropriateness of the figures and tables.	N/A
Please rate the appropriateness of the references.	3
Please evaluate the writing style and use of language.	5 - High/Yes
Please judge the overall scientific quality of the manuscript.	3
Are you willing to review the revision of this manuscript?	Yes

Comments to Author:

Nice presentation of an extremely important issue! Well taken point that no one would end up with the system that is established currently. I like the perspective that a radical consolidation of the surgical scientific societies is of utmost importance. Although - it will never happen, because many people would lack “relevance” within the scientific bodies. Hopefully, some readers will consider some of the arguments that are presented.
